# First successful treatment of epidermolytic Ichthyosis with Vunakizumab: A Case Report

**DOI:** 10.3389/fimmu.2025.1574255

**Published:** 2025-05-16

**Authors:** Wenjie Cheng, Chaolan Pan, Zhe Sun, Peiyi Sun, Jiawen Li, Zhirong Yao, Xiaoxiao Wang, Jia Zhang

**Affiliations:** ^1^ Dermatology Center, Xinhua Hospital, Shanghai Jiaotong University School of Medicine, Shanghai, China; ^2^ Department of Dermatology, Shanghai Jiaotong University School of Medicine, Shanghai, China; ^3^ Institute of Dermatology, Shanghai Jiaotong University School of Medicine, Shanghai, China

**Keywords:** ichthyosis, Vunakizumab, immunotherapy, *KRT1* gene mutation, epidermal barrier dysfunction

## Abstract

Ichthyoses, a group of skin cornification disorders caused by protein and lipid abnormalities that disrupt epidermal functions, are mainly characterized by generalized scaling. This study is the first to report the use of Vunakizumab, China’s first self-developed anti-IL-17A monoclonal antibody, in treating ichthyosis. We presented a case of a 4-year-old boy with epidermolytic ichthyosis (EI) due to a *KRT1* gene mutation. Since birth, he has presented with generalized erythema, desquamation, and blister formation at the extremities. Then, palmoplantar hyperkeratosis gradually emerged, accompanied by severe pruritus. After a three-month treatment with Vunakizumab, symptoms alleviated significantly and inflammatory factor levels normalized. This not only shows the great potential of Vunakizumab in treating ichthyosis but also paves the way for further research on anti-IL-17A therapies for skin cornification disorders, offering treatment options for ichthyosis patients.

## Introduction

Ichthyoses comprise a heterogeneous group of skin cornification disorders, which are either inherited or acquired. The primary clinical manifestation of ichthyosis is generalized scaling, accompanied by inflammation, skin thickening and erythema (1). All ichthyosis subtypes are associated with protein and lipid abnormalities, resulting in defective keratinocyte differentiation and abnormal epidermal barrier formation. Most types of ichthyoses greatly impair patients’ quality of life. Mainstream treatments for ichthyosis are limited to keratolytics, emollient therapies and retinoids, which only have a certain effect on some symptoms or certain subtypes (2). These limitations have driven the pursuit of more effective treatments. Currently, as biologics have emerged in the field of dermatology, novel therapies including Secukinumab and Ustekinumab are being utilized for the treatment of ichthyosis (3, 4). Therefore, understanding the efficacy of biologics in ichthyosis is urgently needed. Vunakizumab is China’s first approved domestically-developed humanized recombinant anti-IL-17A monoclonal antibody (5). It inhibits the interaction of IL-17A with its receptor. As a result, it prevents the production of downstream chemokine CXCL1, blocks further signal transduction, and alleviates the inflammatory process. In this case report, we detail a child with epidermolytic ichthyosis (EI) due to a *KRT1* gene mutation. After traditional therapies failed, treatment with Vunakizumab led to improved symptoms and normalized inflammatory factors in blood, marking its first-ever use in ichthyosis treatment.

## Case report

**Figure 1:**
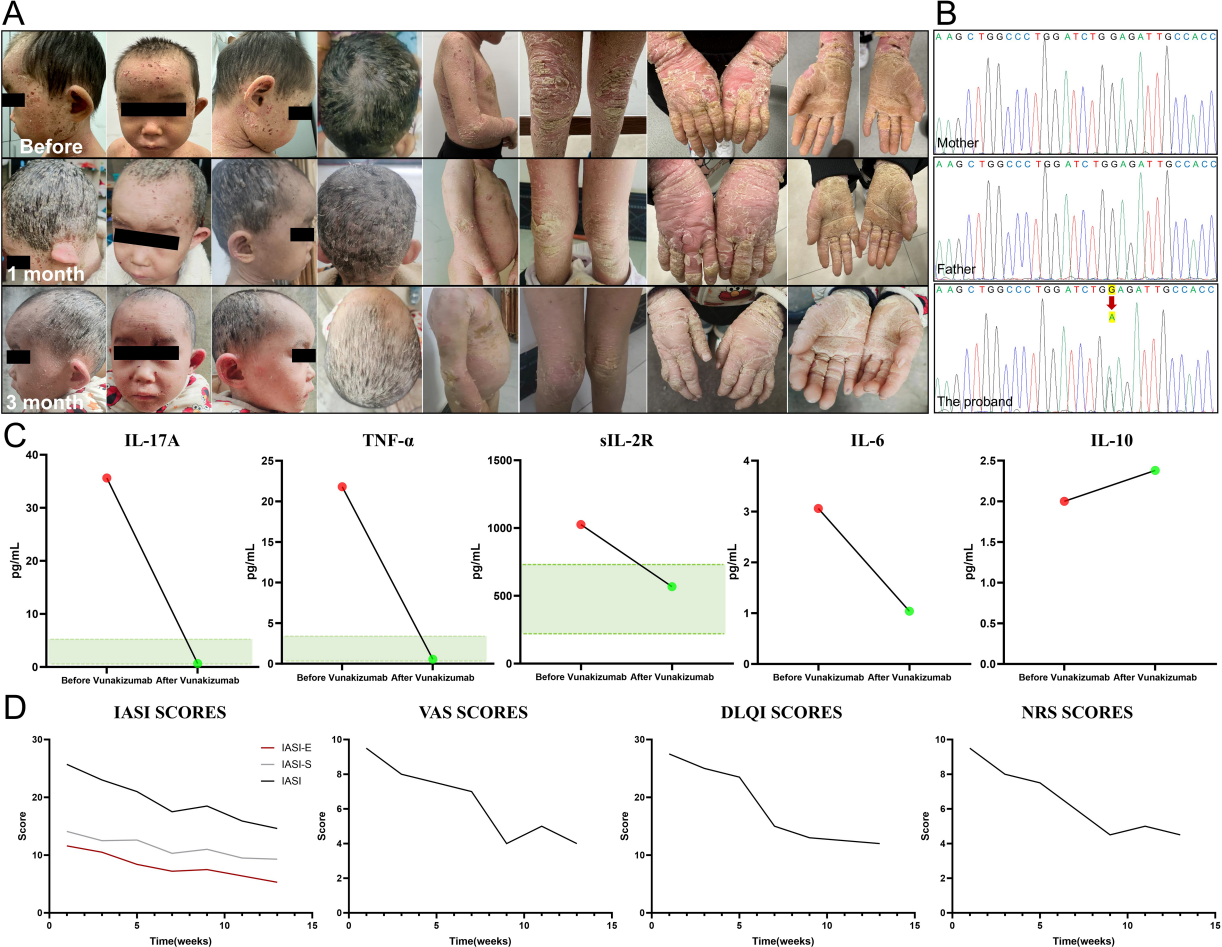
Clinical manifestation, genetic sequencing, inflammatory factor and quantitative score alterations in the patient. **(A)** The patient presented with generalized erythema, desquamation and palmoplantar hyperkeratosis before treatment. After three months of Vunakizumab treatment, remarkable improvements were observed, with alleviated inflammation, diminished erythema, and relieved itching. The keratosis and desquamation also showed a certain degree of amelioration. **(B)** A *de novo* mutation c.1432 G>A (red arrow) of Keratin 1 (*KRT1*) gene was detected in the patient. **(C)** After three months of Vunakizumab treatment, the inflammatory factors including IL-17A, TNF-α and soluble IL-2R returned to normal (The green area represents the normal value range). The level of IL-10 increased slightly. **(D)** Dynamic monitoring of the Ichthyosis Area Severity Index (IASI), including IASI-E and IASI-S, Numerical Rating Scale (NRS), Dermatology Life Quality Index (DLQI) and Visual Analogue Scale (VAS) scores. The scores declined from the baseline and stabilized from the 10th week, highlighting treatment efficacy.

We identified a case of a 4-year-old boy from a non-consanguineous family. Since birth, he has presented with generalized erythema, desquamation, and blister formation at the extremities. Then, palmoplantar hyperkeratosis gradually emerged, accompanied by severe pruritus (**Figure 1A**). The patient had delayed development and no other systemic involvement; moreover, there was no family history of similar diseases. Next-generation sequencing was performed to uncover the root cause of the symptoms. The results, further verified by Sanger sequencing, identified a *de novo* mutation at the locus of *Keratin 1* (*KRT1*) gene [NM_006121.3: c.1432 G>A (p.E478K)] (**Figure 1B**). PolyPhen-2 analysis predicted this mutation to be “probably damaging” (score=1.0), strongly supporting its classification as a pathogenic variant. This Glu478Lys substitution has been previously reported to alter the side-chain net charge from negative to positive and disrupt keratin filament assembly in epidermolytic ichthyosis (EI) patients (6). Combined with clinical manifestations, this genetic finding confirmed the diagnosis of EI (7). When traditional therapies failed to achieve satisfactory results, we conducted tests on the patient’s inflammatory state in blood. Examination showed that the levels of IL-17A, TNF-α and soluble IL-2R were significantly elevated.

Subcutaneous administration of Vunakizumab, China’s first indigenously developed IL-17A antagonist, was chosen as the treatment option for the patient. The treatment was initiated with a dosage of 120 mg every 10 days for three consecutive cycles, followed by a maintenance dosage of 120 mg every three weeks. The three-month follow-up presented marked improvement, including alleviated inflammation and scaling, erythema and itching. The cutaneous keratinization and desquamation demonstrated a certain degree of improvement, yet the improvement was not prominent in the short term(**Figure 1A**). The levels of inflammatory factors, namely IL-17A, TNF-α, and soluble IL-2R, all reverted to normal, while the level of IL-10 increased slightly (**Figure 1C**). In our patient, after using Vunakizumab, the scores of the Ichthyosis Area Severity Index (IASI), including IASI-E (Erythema) which decreased from 11.6 to 5.3 and IASI-S (Scaling) which decreased from 14.1 to 9.3, along with the scores of the Numerical Rating Scale (NRS), Dermatology Life Quality Index (DLQI) and Visual Analogue Scale (VAS), were dynamically monitored (Supplementary Material 1). The scores declined from the baseline and then remained stable from the 10th week (**Figure 1D**).

## Discussion

Keratin 1, encoded by the *KRT1* gene, is a crucial pathogenic gene for EI. It serves as one of the main components of the keratinocyte intermediate filament cytoskeleton (7). *KRT1* dysfunction impairs suprabasal epidermis structural integrity, resulting in skin fragility, blistering, and hyperkeratosis. EI patients exhibit relatively high IASI-S scores, with 78% facing daily life restrictions due to treatment requirements, leading to a poor quality of life (8). Those with *KRT1* mutations have distinct thick palmoplantar keratoderma, prone to fissuring and pain, which limit physical function when severe (9). Moreover, they endure a heavy disease burden, such as heat intolerance, pruritus, and recurrent skin infections. The conserved glutamic acid residue at codon 478 (p.Glu478) of *KRT1*, has a dual role in the structure of keratin intermediate filaments (KIF) (6, 10): participating in initiating structure formation to confer special stability to double-stranded coiled-coil molecules and contributing to stabilizing the bimolecular hierarchical stage in the KIF structure. Multiple missense mutations at codon 478 (p.Glu478) are linked to EI. The p.Glu478Gln (11), p.Glu478Lys (6), and p.Glu478Gly (12), mutations lead to severe phenotypes due to the change in the net charge of the side chain, while the p.Glu478Asp (13–15) mutation retains a negative charge and shortens the side chain by only one carbon atom, causes less damage to keratin filament assembly and results in a milder phenotype.

Traditional ichthyosis treatments have limited efficacy. Emollients, a common choice, are suitable for most patients but time-consuming to apply, resulting in reduced compliance. Additionally, increased skin permeability may heighten the risk of allergic contact dermatitis, especially in warmer climates. Keratolytics are beneficial for patients with thick scaling or hyperkeratosis, have restricted usage and dosage in children. Systemic therapy with oral retinoids can provide further improvement, especially for ichthyosis with obvious skin thickening, but carry risks like teratogenesis, mucosal dryness, and blood abnormalities, making them contraindicated for pregnant women (2, 4). When considering the genetic aspects in EI, it’s worth emphasizing that, EI patients with *KRT1* mutations fare much worse than those with *KRT10* mutations, highlighting the critical need for novel targeted therapeutic innovations for EI patients with *KRT1* mutations (16).

Notably, all ichthyosis subtypes showed marked increases in the expression of IL-17/TNF-α genes. This elevation is consistently observed in both skin and blood samples and is significantly correlated with the overall and erythema severity of the disease (17–20). These findings suggest systemic Th17 activation, thus strongly supporting the application of IL-17 antagonists in systemic ichthyosis treatment. Furthermore, the unique cutaneous microenvironment, predominantly composed of keratinocytes and resident immune cells, may independently modulate IL-17 production and activity. This autonomous regulatory mechanism contributes to the intricate immunological responses observed in ichthyosis. IL-17, as a key cytokine in the Th17 pathway, exacerbates the inflammatory response in EI patients, causing epidermal thickening and immune cell infiltration, consistent with our patient observations. Given that Th17 pathway overactivation drives EI pathogenesis, blocking IL-17 can disrupt the pro-inflammatory cascade. This action mitigates inflammation and erythema, curbs keratinocyte hyperproliferation, restores normal epidermal thickness, and alleviates hyperkeratosis and scaling, ultimately promoting skin normalization (21–24). After being treated with Vunakizumab, the inflammatory factors IL-17A, TNF-α, and sIL-2R levels of our patient normalized, while IL-10 slightly increased. By blocking the Th17 pathway, Vunakizumab significantly reduced the production of pro-inflammatory cytokines, including TNF-α (25). Although the exact function of sIL-2R remains unclear, its downregulation indicates improved disease status (26). Moreover, Vunakizumab enhanced Treg cell differentiation, and the secreted IL-10 modulated the immune response, alleviating inflammation.

This treatment strategy holds particular promise for EI patients with *KRT1* gene mutations. However, clinical evidence regarding the efficacy of IL-17 inhibitors in ichthyosis treatment is inconsistent. While Secukinumab (an IL-17A monoclonal antibody) demonstrated efficacy in congenital ichthyosiform erythroderma (27), a small-scale randomized controlled trial showed that most ichthyosis patients experienced no significant improvement after Secukinumab treatment (28). Notably, two EI patients self-reported symptomatic relief and maintained treatment despite unchanged IL-17 biomarkers, though small sample sizes limit interpretation. In addition, another observational data further reveals differential responses-patients with lamellar ichthyosis or EI exhibited poor biological responsiveness versus improved outcomes in severe syndromic cases (29). Overall, responsive patients reported reduction in daily care time and alleviated pruritus/pain.

Our study demonstrates that IL-17A inhibitor therapy effectively ameliorates ichthyosis through dual suppression of inflammatory pathways and keratinocyte hyperproliferation, with no observed adverse effects or elevated infection rates. This contrasts with previous concerns regarding immune modulation-related infectious risks. Ichthyosis patients are highly susceptible to skin infections due to impaired skin barrier function and immune dysregulation (1). The damaged barrier, along with skin manifestations like excessive keratin production and delayed desquamation, promotes microbial colonization, hinders drug penetration, and causes recurrent infections and clearance difficulties (30). Clinical observations reveal subtype-specific risks: EI patients often have a distinctive body odor, indicating microbial colonization, while infants with Netherton syndrome face life-threatening septicemia risks. EI patients’ skin microbiome differs less than other subtypes; lamellar, EI, and Netherton syndrome patients show increased bacterial abundance, especially *Staphylococcus* and *Corynebacterium*, with decreased fungi except for increased *Trichophyton* in ichthyosis samples (31). Therapeutic drugs targeting the IL-17 family effectively reduce inflammation, are generally safe and well-tolerated. Most related research focuses on psoriasis patients. Meta-analyses show that in adult psoriasis patients anti-IL-17 treatment does not increase the short-term risk of severe infections, and the long-term incidence of severe infections remains low, with no evidence of overall risk escalation from extended use (32). Although it may trigger specific infections like nasopharyngitis and *Candida* infections, the risk increase is slight. Close monitoring during treatment is essential. For ichthyosis patients with atypical manifestations such as hair loss, papules, pustules, increased scaling in summer, and brittle or damaged nails, fungal infections should be suspected. Fortunately, most of these infections are mild to moderate, non-systemic, and can be effectively treated with antifungal agents, rarely leading to patient withdrawal from trials. For ichthyosis treatment, restoring skin barrier function is key to preventing infections.

Vunakizumab, China’s first approved domestically developed anti-IL-17A humanized monoclonal antibody, has been proven to be efficacious for moderate-to-severe plaque psoriasis and was approved in China in August 2024 for treating adult patients with moderate-to-severe plaque psoriasis (5, 33). Given the similar immunological dysregulation involving the IL-17 pathway in both psoriasis and ichthyosis, Vunakizumab’s potential application in ichthyosis has drawn growing interest. Vunakizumab provides several clinical advantages. Vunakizumab acts quickly, with high-dose regimens yielding especially notable results (34), and offers longer dosing intervals, improving patient compliance (35). With a tinea infection rate similar to or lower than Ixekizumab and Secukinumab, it balances anti-inflammatory benefits with a manageable infection risk (33). In the present patient with *KRT1*–related EI, Vunakizumab treatment effectively regulated Th17-mediated inflammation and alleviated skin barrier dysfunction. During the three-month follow-up, patients showed substantial improvements. Inflammation, scaling, erythema, and pruritus significantly decreased, enhancing pediatric patients’ quality of life and reducing daily skin care time. Although skin keratinization and desquamation improved, the changes were less remarkable in the short term. Notably, this is the first report on using Vunakizumab to treat ichthyosis, suggesting its promising potential. However, the study has limitations. The single-case study with a short follow-up restricts evaluating long-term efficacy, safety, and representativeness. The inability to measure TEWL impedes assessing skin barrier impact, and subjective scoring needs objective validation. Thus, while the case report offers initial insights, further large-scale, long-term real-world studies are essential to fully assess its effectiveness and safety.

## Conclusion

In conclusion, this case report details the successful treatment of a child with epidermolytic ichthyosis using Vunakizumab, China’s first domestically-developed and approved anti-IL-17A monoclonal antibody. After treatment, the patient’s desquamation, erythema, and itching symptoms were significantly alleviated, and inflammatory factors normalized, demonstrating the drug’s remarkable efficacy. As the first such report, it highlights Vunakizumab’s potential, offering a new treatment option for ichthyosis and paving the way for further research on anti-IL-17A therapies in skin disorders.

## Data Availability

The raw data supporting the conclusions of this article will be made available by the authors, without undue reservation.
